# The ZBTB24-CDCA7 axis regulates HELLS enrichment at centromeric satellite repeats to facilitate DNA methylation

**DOI:** 10.1007/s13238-019-00682-w

**Published:** 2020-01-22

**Authors:** Swanand Hardikar, Zhengzhou Ying, Yang Zeng, Hongbo Zhao, Bigang Liu, Nicolas Veland, Kevin McBride, Xiaodong Cheng, Taiping Chen

**Affiliations:** 1 Department of Epigenetics and Molecular Carcinogenesis, The University of Texas MD Anderson Cancer Center, Smithville, TX 78957 USA; 2 Program in Genetics and Epigenetics, The University of Texas MD Anderson Cancer Center UTHealth Graduate School of Biomedical Sciences, Houston, TX 77030 USA; 3 Shanghai Key Laboratory of Female Reproductive Endocrine Related Diseases, Obstetrics and Gynecology Hospital, Fudan University, Shanghai, 200011 China

**Dear Editor,**

Immunodeficiency, centromeric instability, and facial anomalies (ICF) syndrome is a rare autosomal recessive disorder characterized by antibody deficiency, facial dysmorphism, failure to thrive, and mental retardation. Patients with ICF syndrome suffer from recurrent and often fatal infections in early childhood. A hallmark of ICF syndrome is loss of DNA methylation in special genomic regions, most notably satellite repeats at centromeric regions, which leads to heterochromatin decondensation and chromosomal abnormalities in lymphocytes (Ehrlich et al., 2008).

The genetic defects of ICF syndrome are heterogeneous. Approximately 50% of cases (ICF1) carry mutations in *DNMT3B* (DNA methyltransferase 3B) (Hansen et al., 1999; Okano et al., 1999; Xu et al., 1999), another ~30% of cases (ICF2) carry mutations in *ZBTB24* (zinc finger- and BTB domain-containing 24) (de Greef et al., 2011), and small numbers of cases carry mutations in *CDCA7* (cell division cycle associated 7) (ICF3), *HELLS* (helicase, lymphoid-specific) (ICF4) or unknown gene(s) (ICFX) (Thijssen et al., 2015). As a major *de novo* DNA methyltransferase, DNMT3B plays an important role in the establishment of DNA methylation patterns during development (Okano et al., 1999). HELLS [also known as lymphoid-specific helicase (LSH)], a SNF2 family DNA helicase involved in chromatin remodeling, has also been shown to regulate DNA methylation, likely by affecting the accessibility of the DNA methylation machinery to genomic regions (Dennis et al., 2001; Ren et al., 2015; Zhu et al., 2006). However, little is known about the biological functions of ZBTB24 and CDCA7 and, in particular, their links to DNA methylation.

Contrary to a recent report by Thompson et al. that ZBTB24 and DNMT3B form a complex (Thompson et al., 2018), we were not able to detect any interaction between ZBTB24 and DNMT3B or DNMT3A with reciprocal co-immunoprecipitation (co-IP) assays using ectopically expressed proteins in HEK293 cells or endogenous proteins in mouse embryonic stem cells (mESCs) (Fig. S1). As DNMT3B is tightly associated with chromatin, its “stickiness” often produces false positive results in protein-protein interaction experiments. Our data suggest that it is unlikely that ZBTB24 directly recruits DNMT3B to genomic regions as Thompson and colleagues proposed. Recently, we and others demonstrated that ZBTB24, a zinc finger (ZF) transcription factor, positively regulates *Cdca7* expression by directly binding a sequence in the *Cdca7* promotor (Ren et al., 2019; Thompson et al., 2018; Wu et al., 2016). We therefore hypothesized that ZBTB24 indirectly modulates DNA methylation via CDCA7.

To test the hypothesis, we first generated mESC lines deficient for *Zbtb24* or *Cdca7* using CRISPR/Cas9-mediated gene editing (Figs. S2 and S3). Consistent with its role in inducing *Cdca7* transcription, *Zbtb24* deficiency resulted in severe downregulation of *Cdca7* without affecting the levels of HELLS and DNMTs (Fig. S4). Southern blot analysis of genomic DNA digested with the methylation-sensitive restriction enzyme *Hpa*II demonstrated that minor satellite DNA, located at centromeric regions, is substantially hypomethylated in mESCs deficient for *Zbtb24* or *Cdca7* (Fig. 1A), recapitulating DNA methylation alterations characteristic of ICF syndrome. The impact of *Cdca7* deficiency on DNA methylation was more severe than that of *Zbtb24* deficiency (Fig. 1A), which likely reflected the effects of complete elimination of *Cdca7* function in *Cdca7*^−/−^ cells and downregulation of *Cdca7* expression in *Zbtb24*^−/−^ cells. Dot blot analysis with a 5mC antibody showed no overt changes in the total level of 5mC in *Zbtb24*^−/−^ and *Cdca7*^−/−^ mESCs (Fig. 1B), indicating that ZBTB24 and CDCA7 play no major roles in the regulation of global DNA methylation. We next asked whether restoring CDCA7 level in *Zbtb24*^−/−^ mESCs would be sufficient to rescue DNA methylation. Indeed, *Zbtb24*^−/−^ mESCs stably expressing HA-tagged CDCA7 at levels comparable to endogenous CDCA7 level in wild-type (WT) mESCs (Fig. 1C) showed complete recovery of the methylation level at minor satellite DNA (Fig. 1D). In contrast, overexpression of Myc-tagged ZBTB24 in *Cdca7*^−/−^ mESCs failed to rescue DNA methylation (Fig. S5), indicating that, although ZBTB24 regulates the expression of multiple genes (Ren et al., 2019; Thompson et al., 2018; Wu et al., 2016), its effect on DNA methylation is dependent on CDCA7. Taken together, these data suggest that the ZBTB24-CDCA7 axis regulates the specificity of DNA methylation in mESCs.

**Figure 1:**
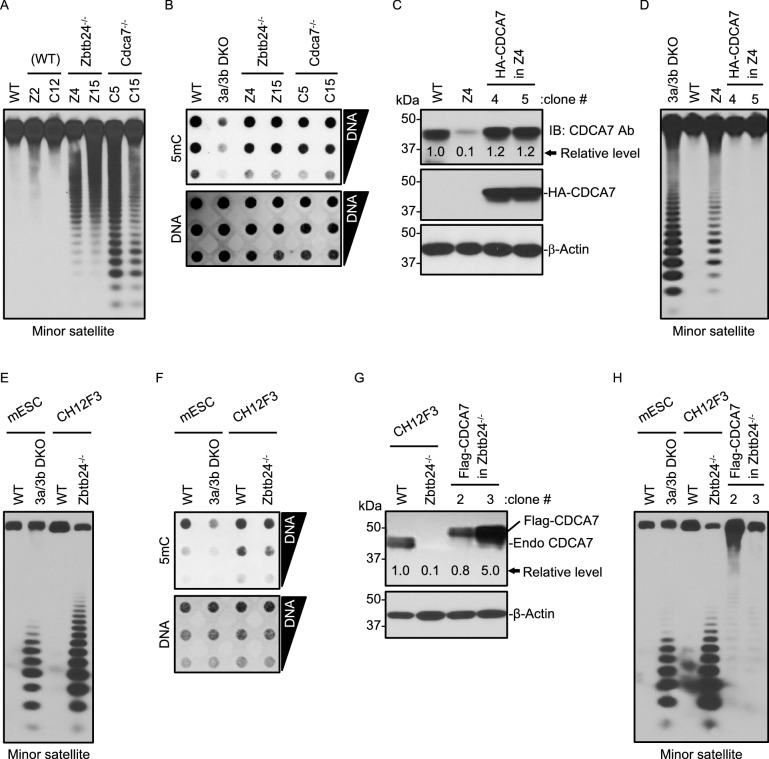
ZBTB24 regulates methylation of minor satellite DNA in a CDCA7-dependent manner. (A) Southern blot showing hypomethylation of minor satellite DNA in mESCs deficient for *Zbtb24* or *Cdca7*, with *Cdca7* deficiency exhibiting more severe effect. (B) Dot blot showing no obvious change in total 5mC level in *Zbtb24*^−/−^ or *Cdca7*^−/−^ mESCs. 3a/3b DKO, *Dnmt3a*/*3b* double KO mESCs. (C) Western blot showing the expression of HA-CDCA7 in stable clones generated in *Zbtb24*^−/−^ (Z4) mESCs. (D) Southern blot analysis of the samples in (C) showing that expression of HA-CDCA7 in *Zbtb24*^−/−^ mESCs results in recovery of DNA methylation at the minor satellite repeats. (E) Southern blot showing hypomethylation of minor satellite DNA in *Zbtb24*^−/−^ CH12F3 cells. (F) Dot blot showing comparable 5mC levels in WT and *Zbtb24*^−/−^ CH12F3 cells. (G) Western blot showing expression of Flag-CDCA7 in *Zbtb24*^−/−^ CH12F3 cells. Both endogenous (Endo) and Flag-tagged CDCA7 are indicated. (H) Southern blot showing that expression of Flag-CDCA7 in *Zbtb24*^−/−^ CH12F3 cells rescued DNA methylation at the minor satellite repeats. Relative protein levels in (C and G) were quantified by densitometry using ImageJ, normalized against β-actin

To determine whether the results obtained in mESCs could be extrapolated in lymphocytes, we disrupted *Zbtb24* in the murine B lymphocyte cell line CH12F3 (Fig. S6) (attempts to disrupt *Cdca7* in CH12F3 cells failed to generate homozygous mutant lines, perhaps because *Cdca7* is essential in these cells). Similar to the effects in mESCs, *Zbtb24* deficiency in CH12F3 cells resulted in drastic downregulation of CDCA7 (*Cdca7* mRNA showed an ~90% decrease and its protein product was hardly detectable) (Fig. S6), DNA hypomethylation at the minor satellite repeats (Fig. 1E), and no obvious effect on total 5mC level (Fig. 1F). Loss of DNA methylation in *Zbtb24*^−/−^ CH12F3 cells was more severe than that in *Zbtb24*^−/−^ mESCs (compare Fig. 1D and 1E). Indeed, methylation of minor satellite DNA in *Zbtb24*^−/−^ CH12F3 cells dropped to levels comparable to those in *Dnmt3a/3b* double knockout (DKO) mESCs (Fig. 1E). The variable effects of *Zbtb24* deficiency in these two cell types were possibly related to different levels of DNMTs and other DNA methylation regulators. Compared to mESCs, CH12F3 cells show lower levels of CDCA7, HELLS, DNMT3A and DNMT3B and also express different DNMT3A and DNMT3B isoforms (Fig. S6). To validate the role of CDCA7 as a downstream effector of ZBTB24 in DNA methylation, we expressed Flag-tagged CDCA7 in *Zbtb24*^−/−^ CH12F3 cells by lentiviral infection, obtaining clones with expression levels that were either similar to or higher than endogenous CDCA7 level in WT CH12F3 cells (Fig. 1G). The methylation levels of minor satellite DNA were largely or fully recovered in these clones (Fig. 1H). Collectively, our results indicate that the role of the ZBTB24-CDCA7 axis in modulating DNA methylation in specific genomic regions, including centromeric satellite repeats, is conserved in mESCs and CH12F3 cells.

While the link between CDCA7 and DNA methylation remains to be determined, recent work revealed that CDCA7 interacts with HELLS (Jenness et al., 2018; Unoki et al., 2019). Given that *Hells*-deficient cells exhibit widespread hypomethylation throughout the genome (Dennis et al., 2001), whereas *Zbtb24* or *Cdca7* deficiency leads to hypomethylation of centromeric satellite DNA without affecting global DNA methylation, we tested the hypothesis that CDCA7 regulates the specificity of the HELLS chromatin-remodeling complex. As shown by chromatin immunoprecipitation (ChIP)-qPCR experiments, HELLS was enriched at minor satellite DNA, and the enrichment was abolished in *Cdca7*^−/−^ mESCs and significantly decreased in *Zbtb24*^−/−^ mESCs and CH12F3 cells that could be restored by ectopic expression of CDCA7. As a negative control, no HELLS enrichment was detected at a region in the *β-Actin* locus (Fig. 2A). ChIP failed to pull down minor satellite DNA from a *Hells*-deficient mESC line, generated by CRISPR/Cas9 gene editing (Fig. S7), thus validating the specificity of the HELLS antibody in this assay (Fig. 2A). Our results demonstrate that CDCA7 is required for the recruitment of HELLS to centromeric heterochromatin.

**Figure 2:**
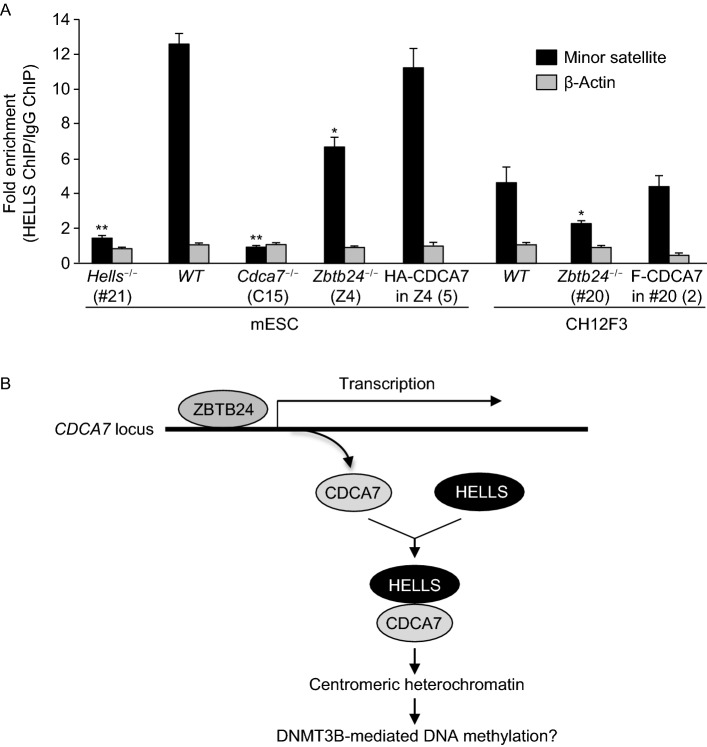
CDCA7 is required for HELLS enrichment at minor satellite DNA. (A) ChIP-qPCR analysis showing that enrichment of HELLS at minor satellite DNA is abolished in *Cdca7*^−/−^ mESCs, significantly reduced in *Zbtb24*^−/−^ mESCs and CH12F3 cells, and rescued in *Zbtb24*^−/−^ cells stably expressing CDCA7. F-CDCA7, Flag-CDCA7. A *Hells*^−/−^ mESC line was used as a negative control. The clone numbers of mutant cell lines used are indicated in parentheses. For each sample, HELLS antibody and IgG were used for ChIP, and qPCR was performed with primers specific for minor satellite DNA or a region at the *β*-*Actin* locus. Shown is fold enrichment (HELLS ChIP over IgG ChIP) for each sample (mean ± SD from two independent experiments). The result of each mutant sample was compared to that of the corresponding WT sample. *P < 0.05; **P < 0.01. (B) Proposed pathway through which the four known ICF-associated genes are involved in the regulation of DNA methylation. ZBTB24 directly activates *CDCA7* transcription, and CDCA7 recruits the HELLS chromatin remodeling complex to centromeric heterochromatin to facilitate DNA methylation by DNMT3B and perhaps other components of the DNA methylation machinery

Loss of DNA methylation in specific genomic regions, including centromeric satellite repeats, is believed to be a primary defect that underlies other cellular and clinical features of ICF syndrome, including centromeric instability and antibody deficiency (Ehrlich et al., 2008). Therefore, elucidating the mechanisms by which DNA methylation is dysregulated is critically important for understanding the molecular pathophysiology of the disease. In this study, we provide genetic evidence that ZBTB24 modulates methylation of minor satellite DNA in a CDCA7-dependent manner in murine cells. We also show that CDCA7 is required for HELLS enrichment at minor satellite DNA. These results define a regulatory pathway for the specificity of DNA methylation, whereby ZBTB24 induces the production of CDCA7, which in turn recruits the HELLS chromatin remodeling complex to special genomic regions, including centromeric heterochromatin, to facilitate the accessibility of the DNA methylation machinery (Fig. 2B). How CDCA7 recruits HELLS to centromeric satellite repeats in a specific manner remains to be determined. All mutations identified in ICF3 cases result in substitutions of conserved amino acids in the CDCA7 C-terminal ZF domain containing four CXXC motifs (Thijssen et al., 2015), and these substitutions do not appear to affect CDCA7-HELLS interaction in our co-IP assays. It is tempting to speculate that the ZF domain of CDCA7 binds centromeric heterochromatin by recognizing a specific genomic or chromatin feature.

## FOOTNOTES

We thank Drs. Tewfik Hamidi and Jiameng Dan for discussions. The ZBTB24 recombinant antibody (V1-36D2-3J2-3) was generated by the Recombinant Antibody Production Core (RAPC) at MD Anderson Cancer Center (MDACC). This work was supported by grants (1R01AI1214030A1 to T.C. and CA16672 to the CCSG Cores at MDACC) from U.S. National Institutes of Health (NIH) and Core Facility Support Awards to MDACC (RP170628 to FCCIC and RP190507 to RAPC) from the Cancer Prevention and Research Institute of Texas (CPRIT). Z.Y. received a fellowship from the Sam and Freda Davis Fund. Y.Z. received a fellowship from the Thomas Endowment. N.V. was supported by a CPRIT Research Training Grant Award (RP170067) and also received a fellowship from the Center for Cancer Epigenetics (CCE) at MDACC and a scholarship from the Andrew Sowell-Wade Huggins Scholarship Fund.

S.H. performed most experiments in mESCs; Z.Y. performed most experiments in CH12F3 cells; Y.Z., H.Z., B.L. and N.V. participated in some experiments; K.M. generated the ZBTB24 recombinant antibody; X.C. participated in experimental design and discussions throughout the study; T.C. conceived the project, supervised the experimental work, and wrote the manuscript.

Swanand Hardikar, Zhengzhou Ying, Yang Zeng, Hongbo Zhao, Bigang Liu, Nicolas Veland, Kevin McBride, Xiaodong Cheng, and Taiping Chen declare that they have no conflict of interest. This article does not contain any studies with human or animal subjects performed by any of the authors.

## Electronic supplementary material

Below is the link to the electronic supplementary material.

Supplementary material 1 (PDF 2013 kb)

## References

[CR1] de Greef JC, Wang J, Balog J, den Dunnen JT, Frants RR, Straasheijm KR, Aytekin C, van der Burg M, Duprez L, Ferster A (2011). Mutations in ZBTB24 are associated with immunodeficiency, centromeric instability, and facial anomalies syndrome type 2. Am J Hum Genet.

[CR2] Dennis K, Fan T, Geiman T, Yan Q, Muegge K (2001). Lsh, a member of the SNF2 family, is required for genome-wide methylation. Genes Dev.

[CR3] Ehrlich M, Sanchez C, Shao C, Nishiyama R, Kehrl J, Kuick R, Kubota T, Hanash SM (2008). ICF, an immunodeficiency syndrome: DNA methyltransferase 3B involvement, chromosome anomalies, and gene dysregulation. Autoimmunity.

[CR4] Hansen RS, Wijmenga C, Luo P, Stanek AM, Canfield TK, Weemaes CM, Gartler SM (1999). The DNMT3B DNA methyltransferase gene is mutated in the ICF immunodeficiency syndrome. Proc Natl Acad Sci USA.

[CR5] Jenness C, Giunta S, Muller MM, Kimura H, Muir TW, Funabiki H (2018). HELLS and CDCA7 comprise a bipartite nucleosome remodeling complex defective in ICF syndrome. Proc Natl Acad Sci USA.

[CR6] Okano M, Bell DW, Haber DA, Li E (1999). DNA methyltransferases Dnmt3a and Dnmt3b are essential for de novo methylation and mammalian development. Cell.

[CR7] Ren J, Briones V, Barbour S, Yu W, Han Y, Terashima M, Muegge K (2015). The ATP binding site of the chromatin remodeling homolog Lsh is required for nucleosome density and de novo DNA methylation at repeat sequences. Nucleic Acids Res.

[CR8] Ren R, Hardikar S, Horton JR, Lu Y, Zeng Y, Singh AK, Lin K, Coletta LD, Shen J, Lin Kong CS (2019). Structural basis of specific DNA binding by the transcription factor ZBTB24. Nucleic Acids Res.

[CR9] Thijssen PE, Ito Y, Grillo G, Wang J, Velasco G, Nitta H, Unoki M, Yoshihara M, Suyama M, Sun Y (2015). Mutations in CDCA7 and HELLS cause immunodeficiency-centromeric instability-facial anomalies syndrome. Nat Commun.

[CR10] Thompson JJ, Kaur R, Sosa CP, Lee JH, Kashiwagi K, Zhou D, Robertson KD (2018). ZBTB24 is a transcriptional regulator that coordinates with DNMT3B to control DNA methylation. Nucleic Acids Res.

[CR11] Unoki M, Funabiki H, Velasco G, Francastel C, Sasaki H (2019). CDCA7 and HELLS mutations undermine nonhomologous end joining in centromeric instability syndrome. J Clin Invest.

[CR12] Wu H, Thijssen PE, de Klerk E, Vonk KK, Wang J, den Hamer B, Aytekin C, van der Maarel SM, Daxinger L (2016). Converging disease genes in ICF syndrome: ZBTB24 controls expression of CDCA7 in mammals. Hum Mol Genet.

[CR13] Xu GL, Bestor TH, Bourc’his D, Hsieh CL, Tommerup N, Bugge M, Hulten M, Qu X, Russo JJ, Viegas-Pequignot E (1999). Chromosome instability and immunodeficiency syndrome caused by mutations in a DNA methyltransferase gene. Nature.

[CR14] Zhu H, Geiman TM, Xi S, Jiang Q, Schmidtmann A, Chen T, Li E, Muegge K (2006). Lsh is involved in de novo methylation of DNA. EMBO J.

