# Supplementary material for: The ZBTB24-CDCA7 axis regulates HELLS enrichment at centromeric satellite repeats to facilitate DNA methylation
Source: Protein Cell. 2020 Jan 22;11(3):214–8. doi: 10.1007/s13238-019-00682-w (PMC7026229; doi:10.1007/s13238-019-00682-w)
Supplement: Supplementary file 1 — Supplementary material 1 (PDF 2013 kb) [file 13238_2019_682_MOESM1_ESM.pdf]

## Materials and Methods

### Plasmid vectors

The Myc- or Flag-tagged DNMT3A and DNMT3B constructs and the *pCAG-Cas9-IRES-GFP* vector were described previously (Hamidi et al., 2018; Kim et al., 2013). The *pL-CRISPR.EFS.GFP* lentiviral vector was purchased from Addgene (Heckl et al., 2014). The Myc-ZBTB24 and HA-CDCA7 constructs were generated by cloning the mouse *Zbtb24* cDNA (GenBank: AAH55367.1) and *Cdca7* cDNA (Accession: NP_080142.1) into the *pCAG-Myc-IRESblast* and *pCAG-HA-IRESblast* vectors (Kim et al., 2013), respectively. The Flag-CDCA7 lentiviral construct was generated by cloning the *Cdca7* cDNA into the *pCDH-EF1-FHC* vector (Yousefzadeh et al., 2014). Primers and oligonucleotides used in this study are listed in Table S1.

### Cell lines

Culture of J1 mESCs and generation of stable clones were described previously (Chen et al., 2003; Dan et al., 2017; Kim et al., 2013; Veland et al., 2017). HEK293 cells were maintained in DMEM supplemented with 10% FBS, 2 mM L-glutamine (Gibco), and 0.5% penicillin-streptomycin. CH12F3 cells were maintained in RPMI1640 supplemented with 10% FBS, 0.05 mM 2-mercaptoethanol, 2 mM L-glutamine, 1 mM sodium pyruvate (Gibco), and 0.5% penicillin-streptomycin. Transfection of mESCs and HEK293 cells was performed using Lipofectamine 2000 (Invitrogen). Infection of CH12F3 cells with lentiviruses was performed using standard procedures. For stable expression of proteins, infected cells were selected with 0.8 μg/mL of puromycin for 7 days, and single cells were seeded into a 96-well plate to derive pure clones.

### Co-IP and Western blotting

Co-IP was performed using either HEK293 cells co-transfected with plasmid vectors expressing Myc- and Flag-tagged proteins or J1 mESCs for endogenous protein-protein interactions. Cell lysates containing 1 μg/mL benzonase nuclease were sonicated. After centrifugation, the supernatants were incubated with Myc, Flag, DNMT3B, or ZBTB24 antibody for 2 h, and Protein A/G UltraLink Resin beads (Thermo Fisher) were used for IP. Western blotting was performed using standard procedures. Antibodies used in this study are listed in Table S2.

### Gene editing in mESCs and CH12F3 cells by CRISPR/Cas9

For gene disruption in J1 mESCs, a synthesized gBlock (Integrated DNA Technologies) containing the sequence for U6 promoter-driven sgRNA was co-transfected with the *pCAG-Cas9-IRES-GFP* vector, and GFP-positive cells were sorted 24 h post-transfection and seeded at low density in dishes coated with feeder cells to derive individual colonies. For gene disruption in CH12F3 cells, the specific 20-bp targeting sequence was cloned into the *pL-CRISPR.EFS.GFP* vector to produce lentiviruses expressing both the sgRNA and Cas9. GFP-positive cells were sorted 24 h after infection, and single cells were seeded into a 96-well plate to derive pure clones. Mutant mESC and CH12F3 cell lines were identified by DNA sequencing and verified by Western blotting.

### DNA methylation assays

Southern blot analysis of DNA methylation at the minor satellite repeats and dot blot analysis of total 5mC levels were performed as reported previously (Chen et al., 2003; Dan et al., 2017; Kim et al., 2013; Veland et al., 2017). To minimize DNA methylation changes associated with long-

term culture, *Zbtb24*- or *Cdca7*-deficient mESCs were cultured for 10-15 passages, and *Zbtb24*-deficient CH12F3 cells were cultured for 8-10 passages.

**RNA isolation and RT-qPCR**

Total RNA was extracted from cells using TRIzol (Invitrogen). Reverse transcription (RT) was performed using ProtoScript First Strand cDNA Synthesis kit (New England Biolabs) to generate cDNA. RT-qPCR was performed using iTaq Universal SYBR Green Supermix (Bio-Rad) on the ABI 7900 Real-Time PCR system (Applied Biosystems) using specific primers (Table S1).

**ChIP-qPCR**

ChIP assays were performed as previously described (Dan et al., 2017; Veland et al., 2017). Briefly, $4 \times 10^6$ cells were treated with 1% formaldehyde for 10 min at room temperature for chromatin crosslinking, then harvested and sonicated. The chromatin was immunoprecipitated with a rabbit polyclonal HELLS antibody or rabbit IgG (Table S2). The immunoprecipitated DNA was used for qPCR analysis with primers specific for minor satellite DNA or a region at the *β-Actin* locus (Table S1).

**References**

Chen, T., Ueda, Y., Dodge, J.E., Wang, Z., and Li, E. (2003). Establishment and maintenance of genomic methylation patterns in mouse embryonic stem cells by Dnmt3a and Dnmt3b. Mol Cell Biol *23*, 5594-5605.

Dan, J., Rousseau, P., Hardikar, S., Veland, N., Wong, J., Autexier, C., and Chen, T. (2017). Zscan4 Inhibits Maintenance DNA Methylation to Facilitate Telomere Elongation in Mouse Embryonic Stem Cells. Cell Rep *20*, 1936-1949.

Hamidi, T., Singh, A.K., Veland, N., Vemulapalli, V., Chen, J., Hardikar, S., Bao, J., Fry, C.J., Yang, V., Lee, K.A.*, et al.* (2018). Identification of Rpl29 as a major substrate of the lysine methyltransferase Set7/9. J Biol Chem *293*, 12770-12780.

Heckl, D., Kowalczyk, M.S., Yudovich, D., Belizaire, R., Puram, R.V., McConkey, M.E., Thielke, A., Aster, J.C., Regev, A., and Ebert, B.L. (2014). Generation of mouse models of myeloid malignancy with combinatorial genetic lesions using CRISPR-Cas9 genome editing. Nat Biotechnol *32*, 941-946.

Kim, S.J., Zhao, H., Hardikar, S., Singh, A.K., Goodell, M.A., and Chen, T. (2013). A DNMT3A mutation common in AML exhibits dominant-negative effects in murine ES cells. Blood *122*, 4086-4089.

Veland, N., Hardikar, S., Zhong, Y., Gayatri, S., Dan, J., Strahl, B.D., Rothbart, S.B., Bedford, M.T., and Chen, T. (2017). The Arginine Methyltransferase PRMT6 Regulates DNA Methylation and Contributes to Global DNA Hypomethylation in Cancer. Cell Rep *21*, 3390-3397.

Yousefzadeh, M.J., Wyatt, D.W., Takata, K., Mu, Y., Hensley, S.C., Tomida, J., Bylund, G.O., Doublie, S., Johansson, E., Ramsden, D.A.*, et al.* (2014). Mechanism of suppression of chromosomal instability by DNA polymerase POLQ. PLoS Genet *10*, e1004654.

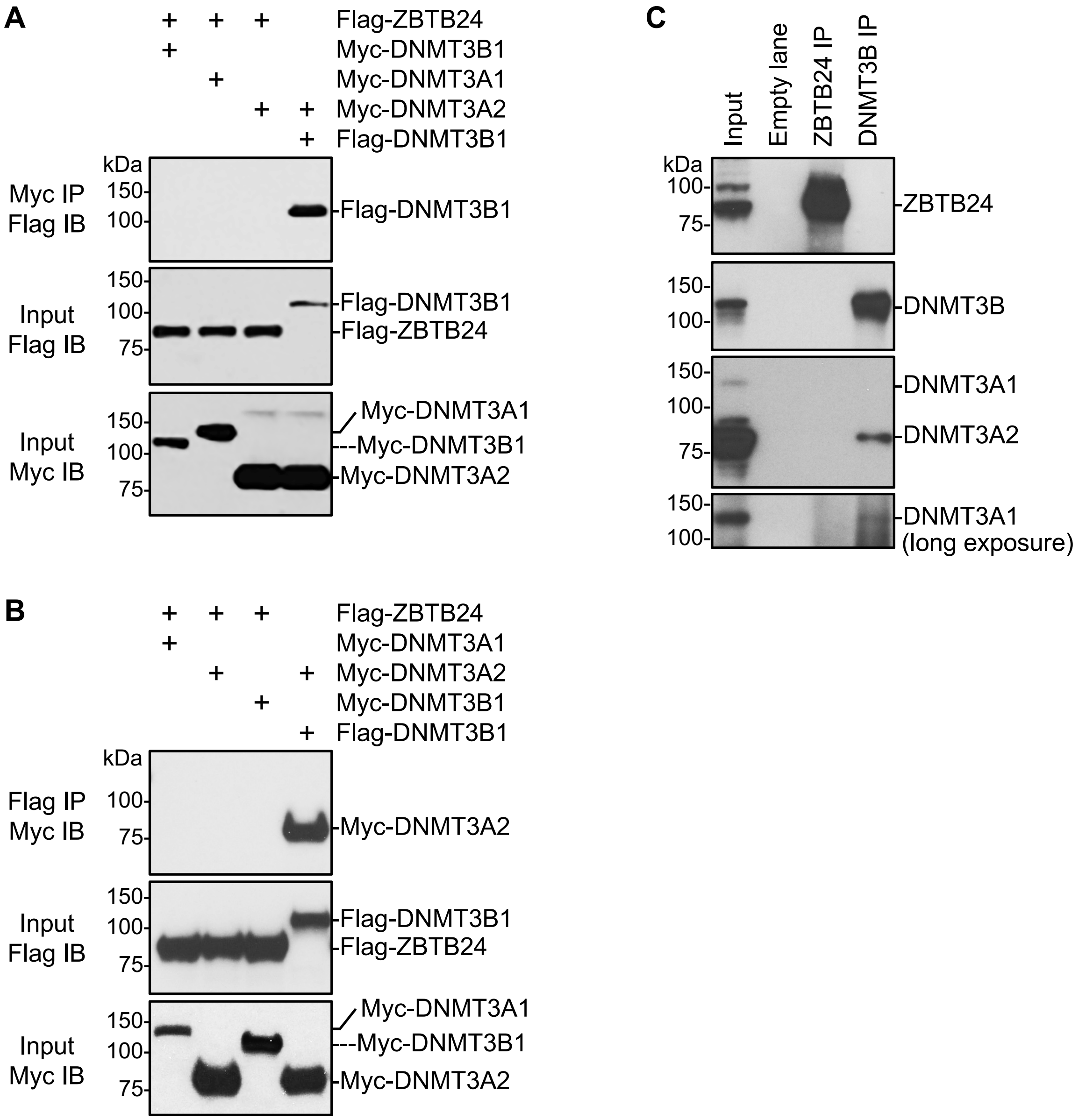


**Figure S1. ZBTB24 does not interact with DNMT3B or DNMT3A.**

**(A, B)** Flag-tagged ZBTB24 and Myc-tagged DNMT3B1 or DNMT3A1/3A2 were co-expressed in HEK293 cells, the cell lysates were used for immunoprecipitation (IP) with Myc antibody **(A)** or Flag antibody **(B)**, and the precipitated proteins, as well as cell lysates (Input), were immunoblotted with Flag or Myc antibody, as indicated. As a positive control, Flag-DNMT3B1 interacts with Myc-DNMT3A2. **(C)** J1 mESC lysates were used for IP with ZBTB24 or DNMT3B antibody, and the precipitated proteins, as well as Input, were immunoblotted with ZBTB24 (PM085), DNMT3B, or DNMT3A antibody. DNMT3B interacts with DNMT3A1 and DNMT3A2, but not with ZBTB24. Note that mESCs express two DNMT3A isoforms: DNMT3A1 at low level and DNMT3A2 at high level. The DNMT3A antibody (CST 49768) does not cross-react with DNMT3B, unlike the one used in Fig. S4 (Abcam 13888), which does.

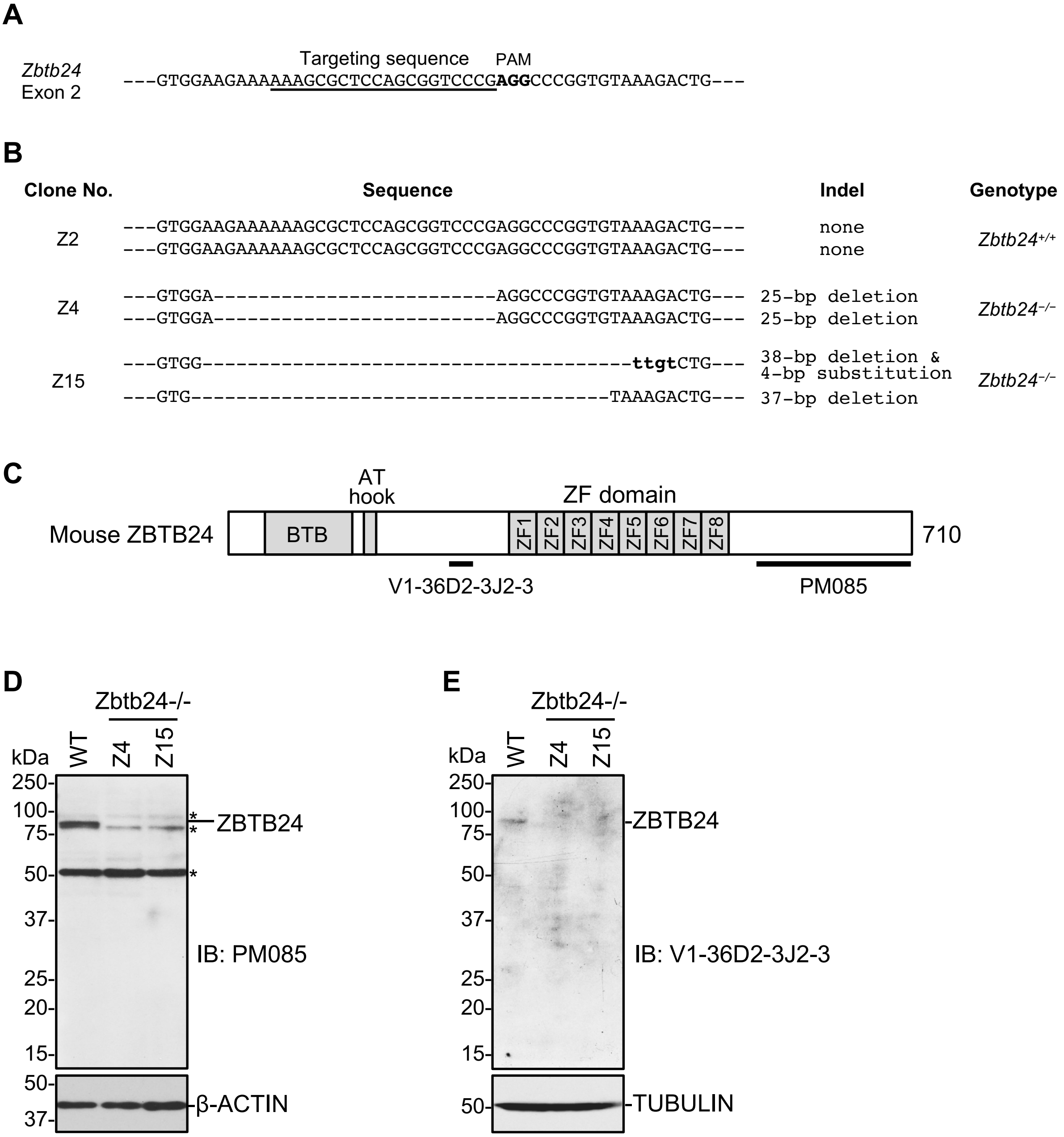


**Figure S2. Generation of *Zbtb24-deficient* mESC lines with CRISPR/Cas9.**

**(A)** Shown is part of exon 2 of *Zbtb24*, with the targeting sequence underlined and the Protospacer Adjacent Motif (PAM) in bold. **(B)** Sequencing results of two null clones (Z4 and Z15), which have frameshift indels on both alleles, and one clone (Z2) with no mutation. **(C)** Mouse ZBTB24 protein is schematically shown, including the BTB domain, AT hook, and zinc finger (ZF) domain with eight tandem ZFs. The regions recognized by the antibodies (V1-36D2-3J2-3 and PM085) are indicated. **(D, E)** Western blots with PM085 **(D)** and V1-36D2-3J2-3 **(E)** showing the absence of ZBTB24 in *Zbtb24*$^{-/-}$ mESC lines Z4 and Z15. Non-specific bands are indicated by asterisks in **(D)**.

**A**

*Cdca7* exon 5 — PAM — Targeting sequence

`---AGAAGGAA`**`CCC`**`GGAACGGAGAACTCGACCTCTTACCAGG---`

**B**

| Clone No. | Sequence | Indel | Genotype |
|---|---|---|---|
| C1 | `---AGAAGGAACCCGGAACGGAGAACTCGACCTCTTACCAGG---` | none | *Cdca7*$^{+/+}$ |
| | `---AGAAGGAACCCGGAACGGAGAACTCGACCTCTTACCAGG---` | none | |
| C5 | `---AGAAGGAACC--------------------------gGG---` | 25-bp deletion & 1-bp substitution | *Cdca7*$^{-/-}$ |
| | `---AGAAGGAACCCGGA-CGGAGAACTCGACCTCTTACCAGG---` | 1-bp deletion | |
| C12 | `---AGAAGGAACCCGGAACGGAGAACTCGACCTCTTACCAGG---` | none | *Cdca7*$^{+/+}$ |
| | `---AGAAGGAACCCGGAACGGAGAACTCGACCTCTTACCAGG---` | none | |
| C15 | `---AGAAGGAACC-------------------TCTTACCAGG---` | 19-bp deletion | *Cdca7*$^{-/-}$ |
| | `---AGAAGGAACC-------------------TCTTACCAGG---` | 19-bp deletion | |

**C**

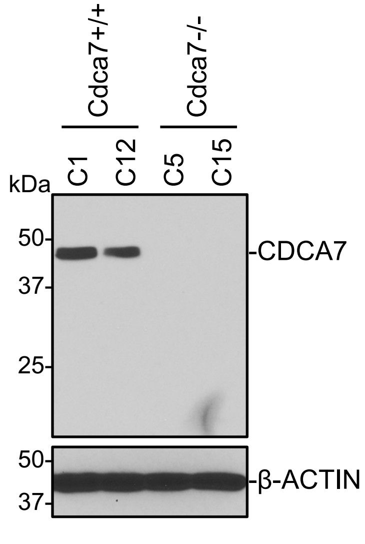


**Figure S3. Generation of *Cdca7*-deficient mESC lines with CRISPR/Cas9.**
**(A)** Shown is part of exon 5 of *Cdca7*, with the targeting sequence underlined and the PAM (reverse orientation) in bold. **(B)** Sequencing results of two null clones (C5 and C15), which have frameshift indels on both alleles, and two clones (C1 and C12) with no mutation. **(C)** Western blot with an antibody against the N terminus (amino acids 1-371 of human CDCA7, Proteintech 15249-1-AP) showing the absence of CDCA7 in *Cdca7*$^{-/-}$ mESC lines C5 and C15.

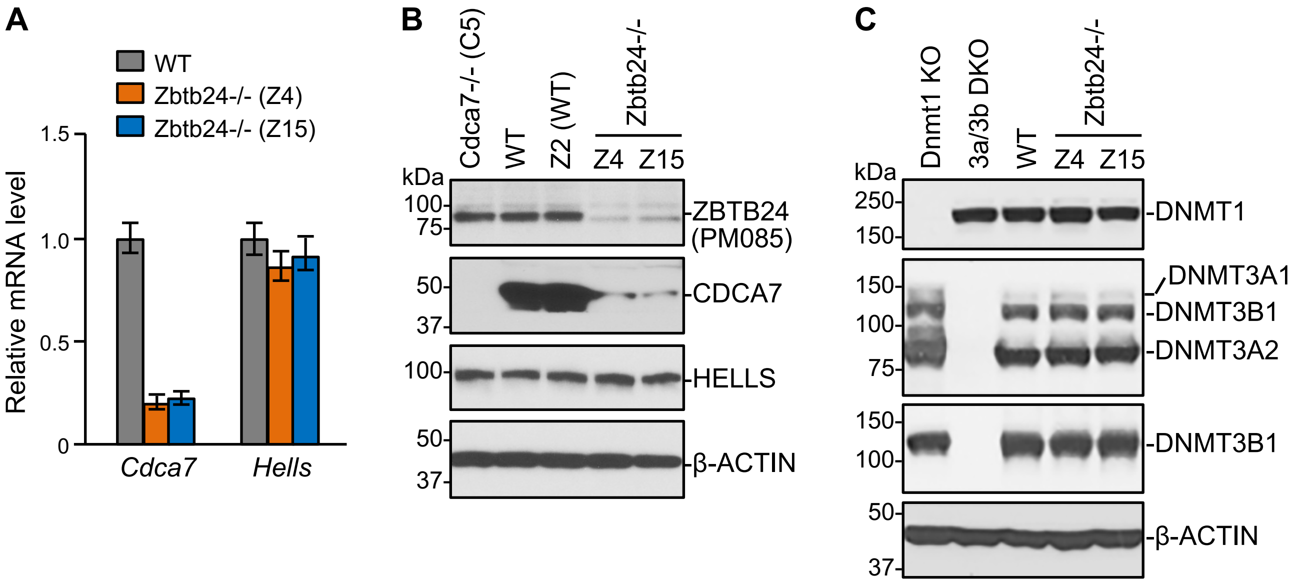


**Figure S4. Severe downregulation of *Cdca7* in *Zbtb24*-deficient mESCs.**
**(A)** RT-qPCR analysis showing downregulation of *Cdca7* mRNA in *Zbtb24*$^{-/-}$ mESCs. **(B, C)** Western blots showing reduced CDCA7 and normal levels of HELLS and DNMT proteins in *Zbtb24*$^{-/-}$ mESCs. Note that the DNMT3A antibody (Abcam 13888) cross-reacts with DNMT3B1, unlike the one used in Fig. S1 (CST 49768), which does not.

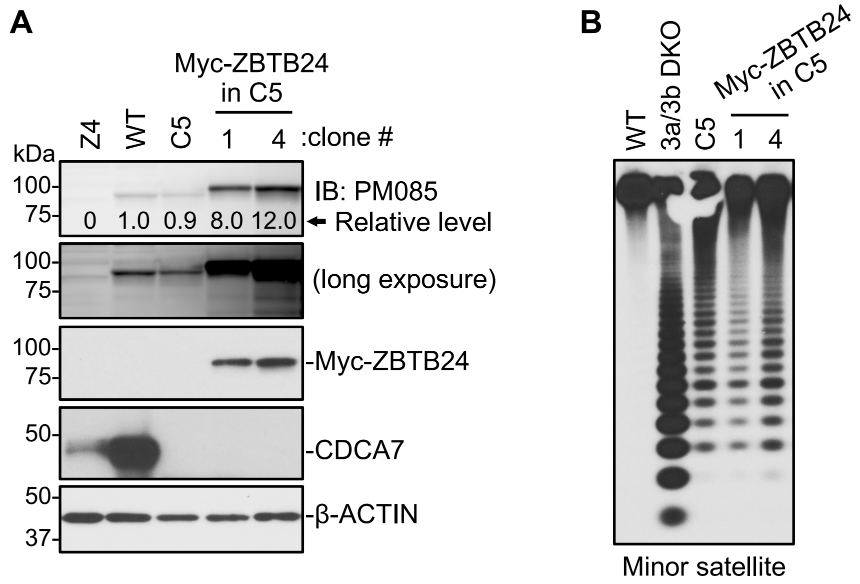


**Figure S5. Overexpression of ZBTB24 in *Cdca7*$^{-/-}$ mESCs fails to rescue DNA methylation.** **(A)** Western blots showing the expression of Myc-ZBTB24 in stable clones generated in *Cdca7*$^{-/-}$ (C5) mESCs. Myc-ZBTB24 levels, relative to endogenous ZBTB24 in WT cells, were quantified by densitometry using ImageJ, normalized against β-ACTIN. **(B)** Southern blot analysis of the samples in **(A)** showing that overexpression of Myc-ZBTB24 in *Cdca7*$^{-/-}$ cells failed to rescue DNA methylation at the minor satellite repeats.

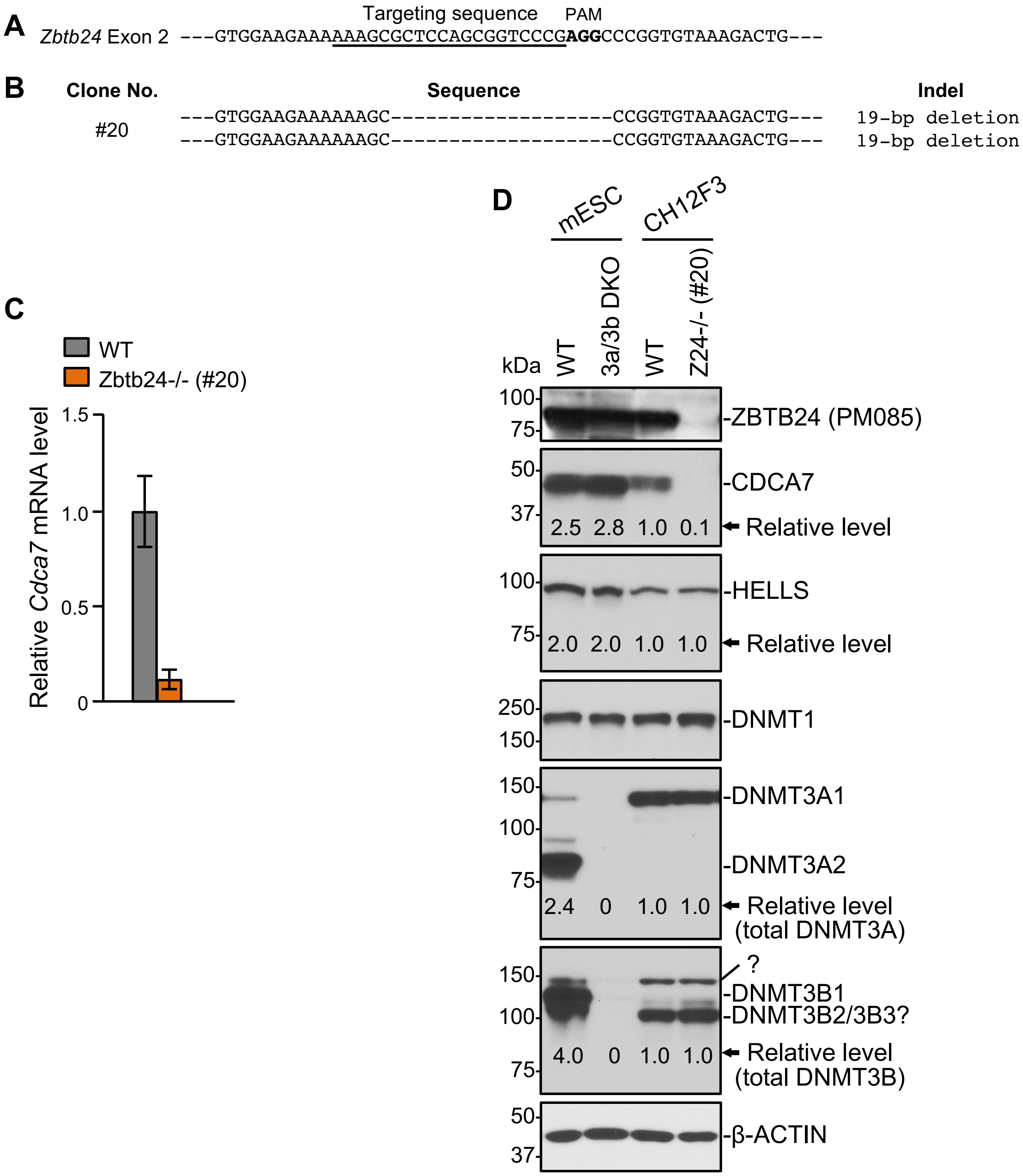


**Figure S6. Generation of *Zbtb24-deficient* CH12F3 cells with CRISPR/Cas9.**
**(A)** Shown is part of exon 2 of *Zbtb24*, with the targeting sequence underlined and the PAM in bold. **(B)** Sequencing result of a null clone (#20), which has a 19-bp deletion on both alleles. **(C)** RT-qPCR analysis showing downregulation of *Cdca7* mRNA in *Zbtb24*$^{-/-}$ CH12F3 cells. **(D)** Western blots showing the levels of ZBTB24, CDCA7, HELLS and DNMTs in WT and *Zbtb24*$^{-/-}$ CH12F3 cells. Relative protein levels were quantified by densitometry using ImageJ, normalized against β-ACTIN. Samples from WT and *Dnmt3a/3b* DKO mESCs were included for comparison. Note that mESCs and CH12F3 cells express different DNMT3A and DNMT3B isoforms: DNMT3A2 and DNMT3B1 were the predominant isoforms in mESCs, whereas only DNMT3A1 and a major DNMT3B band smaller than DNMT3B1 were detected in CH12F3 cells. PCR amplification of cDNAs from CH12F3 cells detected both *Dnmt3b2*, encoding a catalytically active protein, and *Dnmt3b3*, encoding a catalytically inactive protein. Additionally, a weaker band larger than DNMT3B1 was detected in both mESCs and CH12F3 cells, whose identity is unknown

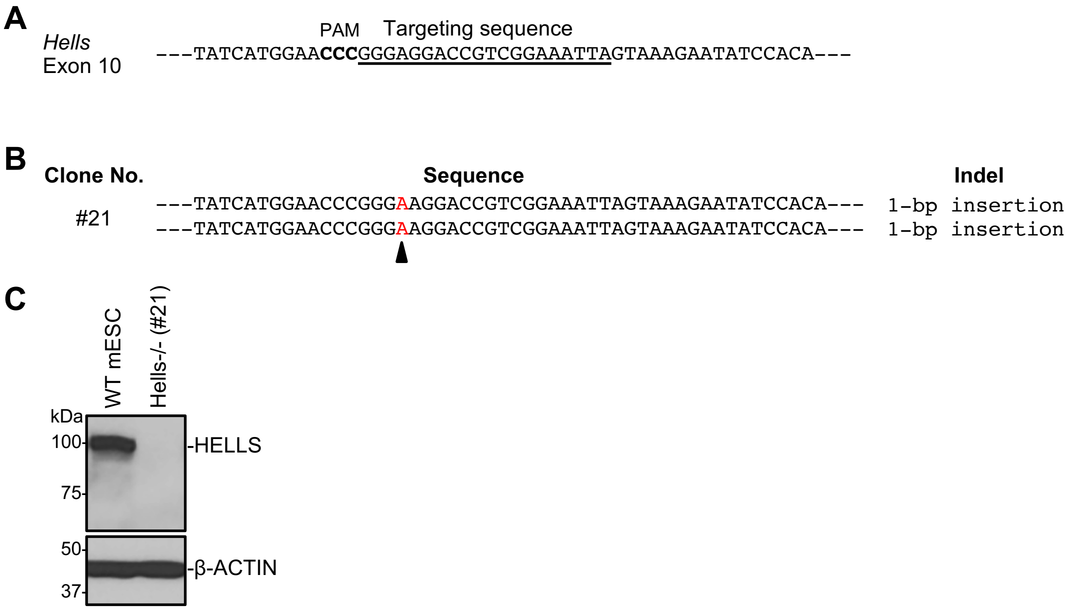


**Figure S7. Generation of a *Hells-deficient* mESC line with CRISPR/Cas9.**

**(A)** Shown is part of exon 10 of *Hells*, with the targeting sequence underlined and the PAM (reverse orientation) in bold. **(B)** Sequencing result of a null clone (#21), which has a 1-bp insertion on both alleles. **(C)** Western blot with an antibody against the N terminus (amino acids 1-348 of human HELLS, Proteintech 11955-1-AP) showing the lack of HELLS protein in clone #21.

## Table S1. DNA primers

| Primers | Sequences (5'-3') | Applications |
|---|---|---|
| C-195(F) | GGA<u>GAATTC</u>GGCAGACACAACCCCGGAG | Amplify *Zbtb24* cDNA |
| C-196(R) | GGC<u>GAATTC</u>ACATCTTTAAGGATCTGTAAG | |
| C-1247(F) | AAC<u>GCGGCCGC</u>TCGCCGCGCGCGGCAAAAG | Amplify *Cdca7* cDNA |
| C-1248(R) | TTC<u>GAATTC</u>TACGCTTGCATTTCAAATTC | |
| C-198(F) | GATGGACAGGAGAGCCAGAG | Screen *Zbtb24* CRISPR clones |
| C-255(R) | CGAGACCTAAACACAACACGAAGG | |
| C-1326(F) | TGCTGGTTCTCTCTGTTACGGAGTA | Screen *Cdca7* CRISPR clones |
| C-1327(R) | AAGCTAAGTGTGTGTGTAGCTCTGA | |
| C-1455(F) | TCCTTATGTTAGTCATGACTTC | Screen *Hells* CRISPR clones |
| C-1456(R) | TTATTGGATGGTAATACTATG | |
| C-1222(F) | CGATGACAGTTGTGACAGCTTTGC | *Cdca7* RT-qPCR |
| C-1223(R) | CGTGCCGGAAACTTCATTGCTAC | |
| C-1226(F) | TCCAGGCTCAAGATAGATGTCATAG | *Hells* RT-qPCR |
| C-1227(R) | GATTGACTTAACCCAGACTGACCAC | |
| C-1525(F) | TGATATACACTGTTCTACAAATCCCGTTTC | ChIP-qPCR for minor satellite |
| C-1526(R) | ATCAATGAGTTACAATGAGAAACATGGAAA | |
| ACTB(F) | TGCCCCGGATCTAGAAAGGA | ChIP-qPCR for $\beta$-*Actin* locus |
| ACTB(R) | TCAGCTGTGGCTGCACATAA | |

F, forward; R, reverse. Restriction sites used for cloning are underlined.

**Table S2. Antibodies**

| Antibody | Vendor & catalog No. | Dilution/Amount | Application |
|---|---|---|---|
| ZBTB24 | MBL Life Science PM085 | 1:2,000 | Western blot |
| ZBTB24 | V1-36D2-3J2-3* | 1:1,000 | Western blot |
| CDCA7 | Proteintech 15249-1-AP | 1:3,000 | Western blot |
| HELLS | Proteintech 11955-1-AP | 1:3,000 | Western blot |
| HELLS | Proteintech 11955-1-AP | 2 μg | ChIP |
| Rabbit IgG | Cell Signaling Technology 2729 | 2 μg | ChIP |
| DNMT1 | Cell Signaling Technology 5032 | 1:1,000 | Western blot |
| DNMT3A | Abcam 13888** | 1:4,000 | Western blot |
| DNMT3A | Cell Signaling Technology 49768*** | 1:3,000 | Western blot |
| DNMT3B | Abcam 13604 | 1:2,000 | Western blot |
| HA tag | Cell Signaling Technology 3724 | 1:5,000 | Western blot |
| Flag tag | Sigma-Aldrich F3165 | 1:5,000 | Western blot |
| Myc tag | Sigma-Aldrich M4439 | 1:5,000 | Western blot |
| β-ACTIN | Sigma-Aldrich A5441 | 1:5,000 | Western blot |
| α-TUBULIN | Cell Signaling Technology 2144 | 1:2,000 | Western blot |
| 5mC | Millipore MABE146 | 1:1000 | Dot blot |

*The ZBTB24 V1-36D2-3J2-3 antibody, generated in-house, is a recombinant antibody against mouse ZBTB24 sequence KDENFDPKAGDGQES. The variable regions correspond to VH (IGHV1-36, IGHD2-3, IGHJ2) and VK (IGKV1-117, IGKJ2) from C57Bl6/J mice. The antibody was recombinantly expressed as a full-length mouse IgG1 in ExpiCHO cells.

**The DNMT3A antibody from Abcam (13888) cross-reacts with DNMT3B1.

***The DNMT3A antibody from Cell Signaling Technology (49768) does not cross-react with DNMT3B1.
